# The Future of Collaborative Human-Artificial Intelligence Decision-Making for Mission Planning

**DOI:** 10.3389/fpsyg.2022.850628

**Published:** 2022-04-04

**Authors:** Sue E. Kase, Chou P. Hung, Tomer Krayzman, James Z. Hare, B. Christopher Rinderspacher, Simon M. Su

**Affiliations:** ^1^U.S. Army Combat Capabilities Development Command – Army Research Laboratory, Aberdeen Proving Ground, MD, United States; ^2^Oak Ridge Affiliated Universities, Oak Ridge, TN, United States; ^3^Department of Computer Science, University of Maryland, College Park, MD, United States; ^4^U.S. Army Combat Capabilities Development Command – Army Research Laboratory, Adelphi, MD, United States; ^5^National Institute of Standards and Technology, Gaithersburg, MD, United States

**Keywords:** decision-making, interface, wargaming and wargames, artificial intelligence, Augmented/mixed reality, visualization

## Abstract

In an increasingly complex military operating environment, next generation wargaming platforms can reduce risk, decrease operating costs, and improve overall outcomes. Novel Artificial Intelligence (AI) enabled wargaming approaches, based on software platforms with multimodal interaction and visualization capacity, are essential to provide the decision-making flexibility and adaptability required to meet current and emerging realities of warfighting. We highlight three areas of development for future warfighter-machine interfaces: AI-directed decisional guidance, computationally informed decision-making, and realistic representations of decision spaces. Progress in these areas will enable development of effective human-AI collaborative decision-making, to meet the increasing scale and complexity of today’s battlespace.

## Introduction

In traditional wargaming, commanders utilize a common map-based operational terrain and model how combinations of factors within the Military Decision Making Process (MDMP, Box 1) produce courses of action (COAs), possible counter-actions, resource usage estimates, and predicted outcomes (Army, 1997, 2014, 2015). Over days or weeks, the MDMP process leads to a refined set of COAs that make certain assumptions about the operating environment, including terrain, weather, and the availability and capabilities of assets in setting the theater (i.e., shaping activity in support of major combat operations).

**Box 1. Military Decision Making Process (MDMP).**

The MDMP is the Army’s doctrinal approach to problem solving starting with receipt of a mission and ending with the production of operational orders. MDMP is used as a tool to assist command staff in examining numerous friendly and enemy COAs. The 7-step process of the MDMP instills thoroughness, clarity, sound judgment, logic, and professional knowledge in the decision-making processes required for planning new missions, extending operations, and performing training exercises (Army, 1997, 2015).

Commanders initiate the MDMP upon receipt of a mission. In Step 1 of the MDMP, all staff and key mission participants are alerted of the mission and the pending planning requirements including the amount of time available for conducting the MDMP. Tools required for performing a mission analysis are identified and documents related to the mission and the area of operations are gathered. Performing the mission analysis, Step 2, builds a full understanding of the mission including critical facts and assumptions resulting in a proposed mission statement and mission analysis briefing in preparation for development of COAs.

Steps 3 through 6 of the MDMP focus on developing COAs for analysis and comparison. These steps include: Step 3, COA development; Step 4, COA analysis (wargaming); Step 5, COA comparison; and Step 6, COA approval. A COA is a potential solution to an identified problem. Each COA is examined for validity using screening criteria such as accomplishing the mission within the established time frame, space, and resource limitations. The COA selection process often involves wargaming, which attempts to visualize the sequential flow of the operation given friendly force’s strengths and enemy’s capabilities while considering the impact and requirements of civilians in the area of operations (Army, 2014). The benefits of a wargaming approach highlight the strengths and weaknesses of the COAs with respect to each other. This tends to be an iterative process where COAs are evaluated and then modified if required until one or more COAs emerge with the highest probability of success for accomplishing mission objectives.

After a specific COA has command approval, the final step of the MDMP is production of the operations order which is a directive to subordinate and adjacent units intended to coordinate the activities of all organizations participating in the mission. This step engages active collaboration among all organizations affected by the disseminated order and builds a shared understanding of the situation.

Although MDMP assists commander staff in understanding an operational environment and considering an operational approach, the process has many limitations such as time intensiveness, rigidity of the assumptions, limited opportunities for training across scenario variations, and few opportunities for integrating Artificial Intelligence (AI) guidance into the decision-making process. Traditionally, the success of a mission is directly related to the ability of command to execute the MDMP. However, given the increased complexity of today’s multi-domain operations (MDO) (Feickert, 2021) with its vast array of mission command systems and processes, integration and synchronization of all activities associated with operations is becoming increasingly difficult to the point of humanly impossible. The lack of planning expertise resulting from a deficient MDMP can lead to desynchronized and dischordant operations and ultimately cost the lives of Soldiers.

The ability to visualize the battlespace is not specifically described in the MDMP, yet it obviously plays an important role in the decision process. Recently, new systems and technologies integrating advanced visualization capabilities have been developed that improve situational awareness and therefore enhance decision-making processes. Army examples include Nett Warrior (Gilmore, 2015), which enables dismounted warriors to visualize nearby friendly and hostile forces while collaboratively planning tactical missions based on the local terrain. Although this technology extends the radio and digital mapping to the dismounted warrior, it lacks an underlying AI engine to provide decision assistance. The Battlespace Visualization and Interaction platform (BVI, formerly Augmented REality Sandtable, ARES) is another example of Army technology that enables distributed collaboration for mission planning with both 2D and 3D visualization capabilities of a common operating picture from arbitrary viewpoints and a wide selection of devices (Su et al., 2021). The BVI architecture is formulated to pull in external computing services such as analytic pipelines, models, and AI engines. Efforts to integrate these types of services into BVI, including AI for enhancing decision support, are underway at the Army Research Laboratory.

Currently, MDMP does not incorporate AI guidance into the overall mission planning approach. The Army’s Automated Planning Framework (APF) (Bailey, 2017) begins to address AI-assistive decision-making by inserting autonomous technologies into the MDMP workflow. Command staff can receive contextual assistance during mission planning and COA development through APF’s digital plan representation, plan creator, and plan monitor tools. Mission execution and estimation capabilities provide automated assistance for improved decision tracking and support activities by monitoring planned versus actual progress of the mission. Although APF introduces a foundational level of automation into the MDMP, it lacks the advanced visualization and user interaction capabilities offered by Nett Warrior and BVI.

Offering both ground force automation and user visualization capabilities is the Army’s most recognized wargaming platform—Semi-Automated Forces (OneSAF) providing modeling and simulation capabilities for computer-generated ground forces (PEO_STRI, 2022). OneSAF offers semi-automated and fully automated modeling of military entities (i.e., soldiers, tanks, helicopters, and aggregate units) in a real-world-like battlespace representation at various levels of fidelity to support specific applications and scenarios. OneSAF is primarily used for training and is interoperable with current mission command systems. It can simulate a wide range of operating environments using multiple-resolution terrains and detailed entity-related databases. However, OneSAF’s advantageous high-fidelity modeling of terrain and entity systems makes it costly to setup and run. It suffers from the limitations of an aging system and is well-known to be difficult to use with experienced soldiers requiring significant training to learn how to operate the simulation (Ballanco, 2019). OneSAF’s complex functionality is not well suited for developing AI-enabled capabilities for rapid and agile warfighter-machine decision-making.

Aside from MDMP and the Army platforms mentioned above, recent efforts to integrate AI into the decision-making process have included a number of approaches (Goecks et al., 2021a), with some success in modeling the human decision-making process. In general, AI has had some success for problems with limited decision variables, such as resource allocation (Surdu et al., 1999), flight simulators (Drubin, 2020), and simpler scenarios. Ongoing challenges include the need to improve the capability of AI to tackle complex decisions with multiple actors, incomplete and possibly conflicting information, changing unit action and environmental properties, and the need to visualize the consequences of these decisions across many spatial and temporal scales and domains.

The following sections describe potential improvements to the MDMP. Section “Required Advancements for Future Military Decision Making Process” overviews three research areas supporting MDO decision-making and graphically depicts the relationships between these research areas and military doctrinal approaches to decision-making. The subsections in section “Required Advancements for Future Military Decision Making Process” offer a more in-depth discussion of each research area. Section “Outlook Toward Advancing Interface Technologies for Human-Artificial Intelligence Team Decision-Making” outlines future directions in the development of warfighter-machine interfaces (WMI) with an emphasis on cross-disciplinary research in human-AI teaming pertaining to decision-making. Section “Conclusion” concludes the paper.

## Required Advancements for Future Military Decision Making Process

Military Decision Making Process limitations to support complex decision-making for MDO highlight the need for improvement in three research areas. First, there is a need to integrate AI generated guidance and assistive decision-making support into the MDMP. This includes both further development and integration of AI into battlespace decision planning, as well as further improvements in the explainability and transparency of the AI’s decision-making process (Chen et al., 2018). Second, there is a need to integrate the decision analytics with the power of high performance computing (HPC), at the strategic level as well as the tactical edge when possible. This would enable leveraging the power of an HPC system to support modeling, analytics, and computation time, while integrating and synchronizing information from across all theater domains. Finally, there is a need to develop more accurate and interactive representations of the decision space using advanced visualization technologies such as mixed reality. Rather than simply displaying a 2D rendering of the terrain at a fixed timescale, there is a need to visualize how decisions across different domains interact, and to exploit mixed reality to both improve the throughput of the understanding and generate insights not possible with flat displays.

**FIGURE 1:**
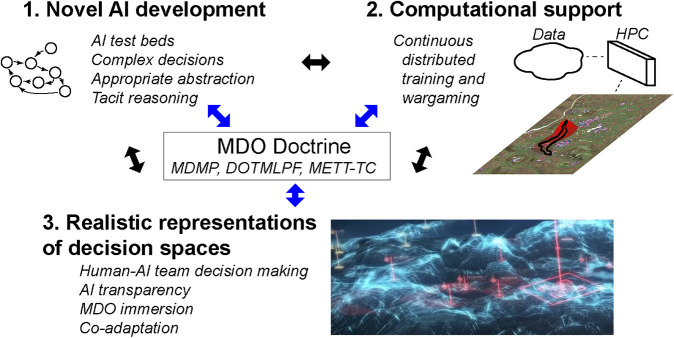
The three research areas of development needed for novel warfighter-machine interfaces (WMIs) and AI-enabled decision aids supporting and enhancing foundational MDO doctrines. [Lower right image source: Lebsack (2021)].

In addition to MDMP, other more broadly applicable military doctrines supporting combative problem solving include: DOTMLPF [e.g., Doctrine, Organization, Training, Materiel, Leadership, Personnel, and Facilities; (Army, 2018)], a framework for identifying gaps and proposing design solutions for current and future warfighting requirements; and METT-TC [e.g., Mission, Enemy, Terrain and Weather, Troops, Time Available and Civil Considerations; (Army, 2019)], a structured framework for capturing the state of mission relevant factors for shared evaluation during military operations. These doctrines define the information context of the MDO battlefield and form a central foundation for military decision-making as applied to the three research areas described above. Research progress and MDO relevant doctrine draw from, inform, and strengthen each other in developing novel representations of complex military decision spaces for both human and AI-enabled command as shown in Figure 1 (Army, 2010).

### Artificial Intelligence-Directed Decisional Guidance

**FIGURE 2:**
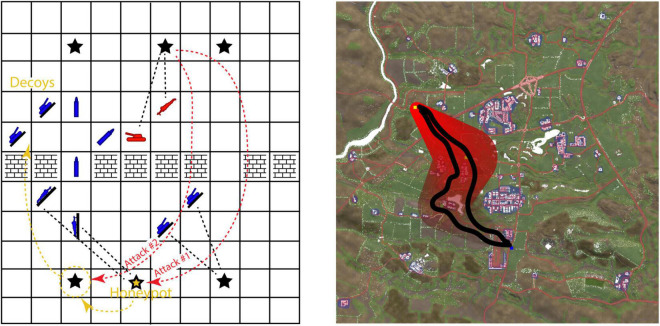
Two example ARL AI testbeds. Left side: ARL Battlespace (Hare et al., 2021) (https://github.com/USArmyResearchLab/ARL_Battlespace). Right side: ARL’s Simple Yeho testbed. Images created by C. Hung.

Novel AI-enabled WMIs are needed to both leverage ongoing advances in AI decision-making and to contribute to AI learning for complex adaptive decision-making. Testing AI decision aids in simplified representations of battlespaces is an important initial step in the development process and a precursor to integrating AIs into more mature battlespace platforms (i.e., BVI, OneSAF). Developing AI testbeds for decision aid experimentation can yield increasingly capable suggestions of possible COAs in MDO. Figure 2 shows two example Army developed AI testbeds.

Artificial Intelligence testbeds enable the development of AIs that pool information across all domains and compute risks and expected rewards for both human and AI agents. The left side of Figure 2 shows the ARL Battlespace testbed (Hare et al., 2021) which is ideal for developing novel AIs for complex decision-making from the ground up. Its abstraction of the battlespace emphasizes core reasoning principles under Army-relevant scenarios, in this case, cyber deception with a honeypot. The smaller grid space enables the AI learning and development to focus on complex reasoning in depth under uncertainty, with multiple friendly and hostile agents. The right side of Figure 2 shows ARL’s Simple Yeho testbed which offers capabilities for integrating AI development with tacit reasoning in more real-world scenarios with multiple terrain-based layers for elevation, viewsheds, obstacles, foliage (concealment), roads, and urban areas. Red shading and black lines indicate the mission start and end points, left and right bounds, and AI-suggested routes. This additional realism enables tie-ins to MDO doctrines including DOTMLPF and METT-TC and enables co-development of AI with naturalistic, opportunistically captured Soldier behaviors. Both of these AI testbeds are extendable as platforms for traditional and immersive mixed reality WMI development.

Use of progressive and extendable AI testbeds allows investigation of several basic limitations of existing AI, particularly for complex and adaptive decision-making with uncertainty, with collaborative and adversarial human and AI agents. Modeling multi-agent collaborative and adversarial decision-making can be particularly complex because of its recursive nature in which other agents are part of the model (Goldman, 1973; Grüning and Krueger, 2021), requiring dynamic and evolving estimates of decision features, individualized values, risk aversion, memory, and attention. These situations of high uncertainty, complexity, and dynamics are areas where humans excel, where appropriately designed interfaces and AI testbeds for human-machine teaming can provide accelerated and more effective decisions. For effective teaming, the novel WMI should help the warfighter to sift through complex information and help the AI to discover implicit rules for decision-making. Below, we provide case examples of how human-machine teaming can be effective.

Complex decision-making as needed in multi-domain wargaming is an immediate challenge for developing effective AI decision aids. The success of recent AIs in games such as Go, Chess, Minecraft, and Monopoly (Silver et al., 2017; Goecks et al., 2021b; Haliem et al., 2021) are based on games with complete knowledge of the existing state of the world (i.e., “open” games), whereas wargaming typically includes incomplete (e.g., Starcraft), uncertain, and/or deceptive information about the operational environment (Vinyals et al., 2019). Uncertainty can also arise from changing physics or other environmental rules, as has been explored with Angry Birds (Gamage et al., 2021). The lack of knowledge makes it difficult for AI agents to calculate the risk-reward profiles of future actions, due to the uncertainty in the state of the world, state of the different actors, and the effects of the actions taken (Cassenti and Kaplan, 2021). Uncertainty also limits the ability of an AI to estimate the risk-reward profiles of the other actors, which are needed to calculate effective game theoretic strategies. It is not uncommon for AI to be overwhelmed by the breadth of possible optimal and near-optimal choices (Lavine, 2019), i.e., selecting the wrong choice due to limited information, since humans employ heuristics to make efficient choices and predictions when developing strategies for effective exploration of hidden information (Gardner, 2019). To assist the development of the AI’s capability for implicit knowledge and exploration, novel WMIs need to explain and present the decision landscape effectively, to allow the warfighter to quickly and naturalistically navigate through possible choices, while enabling the AI to opportunistically learn from human decision-making without imposing cognitive burden (Lance et al., 2020). Such opportunistic learning could include, for example, gaze tracking to capture visual regions and unlabeled targets that attract human interest and intent. They could also include actor critic methods building on naturalistic Soldier choice behaviors, to improve the AI’s learning of how human experts prioritize certain choices under uncertainty, incomplete information, and deception, depending on mission-relevant contexts.

**FIGURE 3:**
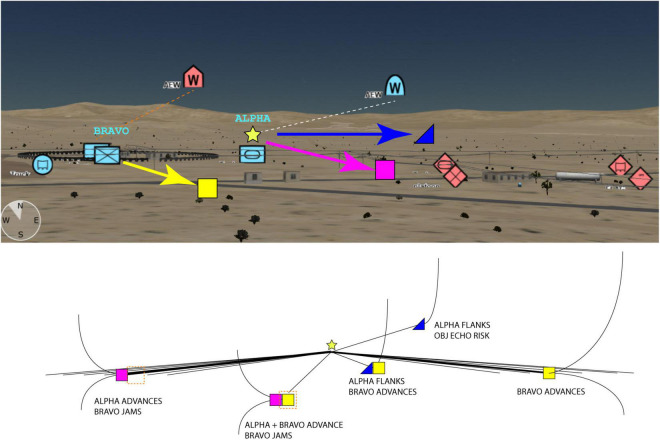
At the top, a 3D view of a friendly vs. hostile wargame scenario in the BVI Web Tactical Planner application. The 3D view offers a more realistic decision-making perspective than a 2D view, for example, showing the elevations of friendly (blue) and hostile (red) Airborne Early Warning systems (AEWs) and the surrounding terrain. This enables rapid review of possible sightlines and sensing relative to the surrounding terrain. Below is the AI’s navigable decision tree, providing transparency to the AI’s calculated risk/reward profiles of a few key choices and how they map onto the terrain. Such abstract decision spaces would also enable integration of non-spatial decisions, e.g., cyber deception. Dashed lines indicate communication links to friendly AEW and possible jamming of hostile AEW. Images created by C. Hung.

Another fundamental challenge for developing AI-enabled WMIs is how to effectively integrate and display information across all five domains in MDO, particularly space and cyber, as information across these domains have disparate spatiotemporal scales (Gil et al., 2018). For cyber, the scale and speed of the decision-making can be faster than human capabilities to process and understand, requiring human input to guide semi-automated decision-making and an AI that implements strategies for offensive and defensive deception. The WMI needs to be able to display the decision landscape in such a way that a small list of optimal and near-optimal decision strategies are explainable (e.g., the decision tree in Figure 3). This should include estimates of the future states and risk-reward profiles of key agents under uncertainty (Hare et al., 2020), to allow effective game theoretic decision-making to be co-developed and mutually understood.

These challenges inform the possible design of effective WMIs. Namely, we need the capability to ingest information from disparate sources (including from other nations’ decision aids) and an architecture that can host the computational power to integrate this information, while also handling the underlying AI computations (both for learning and for deployment). We also need to co-develop an interface and algorithm design that opportunistically harnesses the strengths and mitigates the limitations of human and AI agents.

### Computationally Informed Decision-Making

Substantial computation power is needed to process and record all components, entities and state spaces during complex decision-making. Past, present, and predictive modeling from accumulated datasets of dynamic state spaces requires leveraging HPC resources for generating analytic insights and creating representations useful in decision-making contexts.

One approach for implementing a HPC analytic workflow uses Persistence Services Framework (PSF). PSF is a recently available distributed virtualization solution that enables non-traditional access to HPC services through a web-based front end, unlike traditional HPC environments where computational nodes are allocated to users in batch mode for a specific period of time. Additionally, PSF provides distributed and continuous access to data, databases, containerized toolsets, and other hosted platforms (Su et al., 2021).

In an example PSF approach, a simulation engine connects to PSF for recording all decisions made by both the humans and AIs. This allows analysis of the decision-making behavior occurring during mission planning and COA development, as well as identification of decision-making patterns and strategies for developing competitive and realistic wargaming scenarios. A battlespace visualization platform can be hosted on PSF and use a messaging protocol to update all connected device interfaces. State information from the simulation engine can be used for generating graphical representations of the battlespace and the engaged operational units.

Using a PSF-approach and taking advantage of HPC resources allows implementation of AI-assistive decision-making mechanisms exploiting big data ingests and analytics, while being available to geographically distributed users for collaborative decision-making efforts and “always-on” individualized training and red teaming. A variety of mixed reality display modalities connected to a PSF-hosting server can support a range of operational scenarios from command and control at the strategic level to more mobile tactical use at the operational edge.

### Realistic Representations of Decision Spaces

Graphically representing military decision-making strategies at all levels of operations requires new visualization approaches that can be applied to dynamic environments characterized by changing rules, cognitive states, uncertainty, and individual biases and heuristics (Dennison et al., 2020; Hung et al., 2020; Raglin et al., 2020). The visual representation of a battlespace should be as accurate and realistic as technologically possible, yet remain at a cognitive level that is human understandable and interpretable (Kase et al., 2020; Larkin et al., 2020; Hung et al., 2021). Advanced visualization approaches that incorporate mixed reality technologies have the potential to better represent the changing character of multi-domain warfare and its evolving threats and dynamic environments. With recent technological advancements in mixed reality visualization devices, lowered costs and significantly improved reliability and usability of hardware, hybrid 2D and 3D visualization approaches are now possible.

Mixed reality approaches comprised of multiple 2D monitors augmenting more advanced 3D visualization capabilities can provide command staff with the necessary insights needed to understand complex wargaming state spaces (Su et al., 2021). When a shared battlespace representation is required, a collaborative strategic planning mode can be achieved with multiple coordinated views implemented on different visualization modalities to update interactively based on distributed command staff inputs.

The BVI (Garneau et al., 2018) platform represents geospatial terrain information and map images allowing command staff to build and modify tactical mission plans and COAs. As a data server, BVI distributes terrain and operational data to client applications supporting multiple visualization modalities including Head-Mounted Display devices, web-based interfaces, mobile Android tablet devices, and mixed reality devices (e.g., HoloLens 2, Oculus Quest).

For example, Figure 3 (top) shows a Friendly versus Hostile wargaming scenario on a high-resolution terrain of the Fort Irwin National Training Center located in San Bernardino County, California (Wikipedia, 2021). A 3D view of the battlespace can offer a more enriched user experience from multiple viewing perspectives than the traditional 2D map display often used during MDMP. The 3D view, in BVI’s Web Tactical Planner (WTP) visualizes spatial information of both terrain and man-made features and the positions of the units depicted by MIL-STD 2525C symbols (DOD, 2014). Geospatial perspectives, such as those offered by BVI, conceivably support decision makers’ understanding of dynamic battlespace environments. Paired with a navigable AI-augmented decision space (Figure 3, bottom), the combined perspective can enable better understanding across visual spatial dependencies, effects and causalities, estimated risks and values, uncertainty, and deception for complex decision-making. Combining such geospatial and decision-centric perspectives with AI may provide the necessary breadth to coordinate physical actions with actions in cyber and other non-spatial domains across multiple timescales, as well as the flexibility to adapt quickly to changing mission objectives.

## Outlook Toward Advancing Interface Technologies for Human-Artificial Intelligence Team Decision-Making

Rapid advances in development of AI and human-AI teaming require concurrent advances in the development of WMI. As novel AIs produce better predictions of rewarding COAs and are better able to tackle complex decision-making, they must also leverage human expertise to learn *how* to tackle decisions with high uncertainty, deception, tacit knowledge, and game theory. Conversely, the AI’s reasoning must be both abstracted and relatable to the wargaming environment, to enable transparency and trust without imposing undue cognitive burden. A WMI that is based on 3D mixed reality can harness and augment inherent human capacities for 3D cognition and prediction (Welchman et al., 2005; Kamitani and Tong, 2006; Kim et al., 2014; Boyce et al., 2019; Krokos et al., 2019), and if it is appropriately designed, its interface will feel naturalistic while expanding the capability to display information from across multiple domains while enabling the AI to opportunistically learn from the user’s decision-making.

We have highlighted three key areas for development, namely the AI-directed decision guidance, the computational infrastructure to support this guidance, and the development of mixed reality representations for decision transparency. Advances in these areas require expertise across many different disciplines. Novel AI development requires the fusion of ideas from neuroscience, psychology, and mathematics, to overcome bottlenecks to longstanding problems in complex decision-making. This includes learning across long timescales and catastrophic forgetting under changing contexts, as well as problems more specific to wargaming such as multi-agent decision-making with uncertainty, deception, and game theory. The computational infrastructure also needs development, as computing power and data frameworks are both essential for producing common operating pictures for human-AI teaming at the tactical edge. For efficient development, proprietary restrictions and software dependencies should be abstracted away *via* a common framework, with clear documentation for usage and troubleshooting, to allow academia, government, and industry to better focus on tackling the human-AI teaming problem. This common framework should include efficient passing of information, while providing flexibility and adaptability to the needs of both the AI development and the human user across both training and live-use environments. Finally, the development of the interface itself needs concerted expertise across multiple disciplines. A foundational problem is how to compact information to be efficiently understood by the user, and how to best harness user interactions for opportunistic learning. The human mind does not process all sensory information, but instead makes predictions and assumptions about the world to economize its computations under an environment with incomplete information. An effective WMI should anticipate both potential decision outcomes as well as individual user expectations and assumptions. Additionally, the AI decision aid must estimate what is the user’s tacit understanding, allowing it to present the most relevant information and the most promising choices pulled from across the warfighting domains.

## Conclusion

Information operations and command and control (C2) are two capabilities that the United States Army can provide to allies and partners. In the future operational environment, we must prepare for not only kinetic operations, but also hybrid and information-focused warfare. This requires advances in AI capabilities for complex and tacit reasoning, advances in systems that can provide continuous training, distributed hybrid decision-making, and big data ingestion and analytics, as well as advances in human-AI collaborative decision-making and opportunistic learning for continued AI advancement and human-AI co-adaptation. Each of these advances requires cross-disciplinary programmatic efforts to overcome complex technological challenges and to create new principles, theories, and doctrinal approaches to decision-making, including sustained development of integrative testbeds and technologies to enable collaborative and synergistic development across government, academia, and industry.

## Data Availability Statement

The original contributions presented in the study are included in the article, further inquiries can be directed to the corresponding author.

## Author Contributions

SK created the outline. SK, CH, and TK wrote sections and reviewed manuscript. JH reviewed the manuscript and contributed sections that were integrated in the manuscript by CH and SK. BR and SS reviewed the manuscript. All authors contributed to the article and approved the submitted version.

## Author Disclaimer

The views and conclusions contained in this document are those of the authors and should not be interpreted as representing the official policies, either expressed or implied, of the Army Research Laboratory or the United States Government. Certain commercial equipment, instruments, or materials are identified in this manuscript in order to specify the experimental procedure adequately. Such identification is not intended to imply recommendation or endorsement by the National Institute of Standards and Technology (NIST), nor is it intended to imply that the materials or equipment identified are necessarily the best available for the purpose. The United States Government is authorized to reproduce and distribute reprints for Government purposes notwithstanding any copyright notation herein.

## Conflict of Interest

The authors declare that the research was conducted in the absence of any commercial or financial relationships that could be construed as a potential conflict of interest.

## Publisher’s Note

All claims expressed in this article are solely those of the authors and do not necessarily represent those of their affiliated organizations, or those of the publisher, the editors and the reviewers. Any product that may be evaluated in this article, or claim that may be made by its manufacturer, is not guaranteed or endorsed by the publisher.
